# Association of Salivary Cortisol With Anxiety in Type 2 Diabetes Mellitus Patients Before and After Complete Denture Rehabilitation: An In-Vivo Analysis

**DOI:** 10.7759/cureus.51953

**Published:** 2024-01-09

**Authors:** Akansha Bansod, Sweta G Pisulkar, Surekha A Dubey, Seema Sathe, Arushi Beri, Chinmayee Dahihandekar

**Affiliations:** 1 Prosthodontics, Sharad Pawar Dental College and Hospital, Datta Meghe Institute of Higher Education and Research, Wardha, IND

**Keywords:** dass-21, dental treatment related anxiety, anxiety, geriatric population, type 2 diabetes mellitus, edentulism, complete dentures

## Abstract

Background

Complete edentulism negatively impacts emotional, physical, social, and psychological well-being, leading to a decline in quality of life and heightened stress and anxiety. Stressful situations associated with edentulism can elevate cortisol levels, potentially increasing the risk of diabetes. Rehabilitation with complete dentures needs careful evaluation for its impact on general health, considering stress points and systemic effects. This study aimed to assess salivary cortisol levels in type 2 diabetes mellitus patients before and after complete denture rehabilitation, highlighting the intricate relationship between diabetes, cortisol, and the stress response.

Methods

This is a cross-sectional study centered on individuals with diabetes who were completely edentulous and undergoing evaluation by the outpatient prosthodontic department. Glycated hemoglobin, anxiety levels, and the fabrication of complete dentures were all accomplished with the participants' consent. Patients underwent evaluations before and one month after receiving complete denture rehabilitation.

Results

An absolute correlation between salivary cortisol levels and anxiety may be established because anxiety levels dramatically decreased during complete denture rehabilitation.

Conclusion

By learning how the patient's general health is related to their new set of complete dentures and how to correlate that information with dental rehabilitation, professionals can help patients better adapt to their new set of dentures.

## Introduction

Complete edentulism places a significant load on the world's healthcare system. As per the prosthodontic terminology, edentulism can be defined as being toothless; being edentulous means the absence of any natural teeth [1]. It is the physical state of the jaw(s) after the extraction of all naturally erupted teeth, encompassing the evaluation of available supporting structures for reconstruction and/or replacement therapy, as complete edentulous individuals necessitate treatment [2]. Edentulism remains an irreversible and chronic condition that develops as a result of a multifactorial process that includes biological factors (periodontal diseases, pulpal pathology or caries, trauma, and oral malignancy) and patient-related aspects (access to care, patient preferences, treatment possibilities, and so on) [3]. It reduces the oral health of patients and subsequently affects the quality of life by altering chewing efficiency, nutrition, and general health [4]. Complete tooth loss is accompanied by the same set of known risk variables as any other non-communicable illnesses and lifestyle changes such as smoking, alcohol consumption, and carbohydrate-rich eating habits [5].

Tooth loss, which has a variety of negative anatomic, cosmetic, and biomechanical consequences, can be a devastating psychological blow to sufferers. To provide a comprehensive treatment amongst the edentulous population, one must first understand both the physical and emotional aspects of tooth loss. The term edentulous is frequently connected with the elderly. Stress is a major emotional condition connected with aging [6] The psychological reaction to the loss of teeth and denture wear may be influenced by a patient's temperament or mental condition. Cardiovascular disorders or any systemic events, that actively contribute to vigilance alteration and are subject to neuronal and hormonal effects, can be brought on by stressful situations. These mostly have the effects of increasing heart rate, blood pressure, and immune cell stimulation while also activating the hypothalamus axis, resulting in the release of various hormones, including cortisol, which is particularly known as the stress hormone [7]. The hypothalamic-pituitary-adrenal (HPA) axis serves as the regulatory system for cortisol and other stress-related hormones, often referred to as the body's stress system. The activation of the HPA axis is a natural physiological response to emotional and physical stress, aiming to protect the individual and restore balance (homeostasis) in challenging and stressful situations. In response to stress, the para-ventricular nucleus in the hypothalamus releases corticotrophin-releasing hormone, initiating the cascade of events in the HPA axis [8].

The pituitary gland responds by secreting adrenocorticotropic hormones. In turn, the adrenal gland stimulates and releases hydrocortisone or serum cortisol in the bloodstream. A negative feedback loop makes cortisol a key regulator of the physiologic stress response [9]. Serum, gingival crevicular fluid (serum transudate), and, more recently, saliva, have all been considered as prospective sources for biomarker analysis in stress. According to studies, the cortisol present in saliva represents biologically free active cortisol hormone, not affected by the flow rate of saliva, and accurately and consistently reproduces HPA axis reactivity and free serum cortisol, and also, is a more efficient stress assessment tool compared to vein puncture because it is less likely to cause false increases in cortisol release that indicate a hyper-stress component [10]. Blood glucose levels increase as an outcome of increased cortisol. By amplifying the effects of the adrenaline, it increases glycogenolysis activity in the liver and accelerates gluconeogenesis. As a result, a significant amount of glucose enters the bloodstream in a very short duration [11]. Excess activity of glycogenolysis and gluconeogenesis causes hyperglycemia, favoring the occurrence of systemic disorders like diabetes and other disorders of glucose metabolism [12].

The rising incidence of diabetes is a concerning health trend affecting populations worldwide, and within the next 25 years, it's predicted to rank among the leading causes of death globally [13]. If we add informational indices, India is known as the world's diabetic capital. In comparison to dentate patients, the diabetes risk is 1.82 times higher in edentulous people. With longer lifespans, prosthodontists will certainly see a greater number of diabetic patients [14]. However, the relation of diabetes to edentulism is unknown. Given the conflicting findings of prior studies, it is probable that the link between edentulism and diabetes is contextual, necessitating the collection of more data to detect the potential link between edentulism and diabetes. Therefore, it is imperative to evaluate the correlation between complete denture therapy and the general health of edentulous patients from a functional and aesthetic standpoint. It can be done by measuring stress levels using hormonal markers and evaluating their effect on overall health.

Edentulism and diabetes may have mutually harmful consequences on various domains of health, including insomnia and perceived stress, resulting in elevated cortisol levels, and evaluating the effects is crucial to comprehending the health outcome of this metabolic comorbidity. There is a paucity of studies on edentulous populations suffering from type 2 diabetes mellitus and the correlation to stress and anxiety. Edentulism creates anxiety, and more anxiety leads to stress, which in turn causes uncontrolled diabetes. Consequently, it is important to establish a correlation between these determinants. Ascertaining the need for studying the correlation of the stress biomarkers in edentulous patients with systemic disorders, the present study compares and determines the correlation of anxiety to the cortisol levels in saliva, in diabetes patients rehabilitated with complete dentures.

## Materials and methods

**Figure 1:**
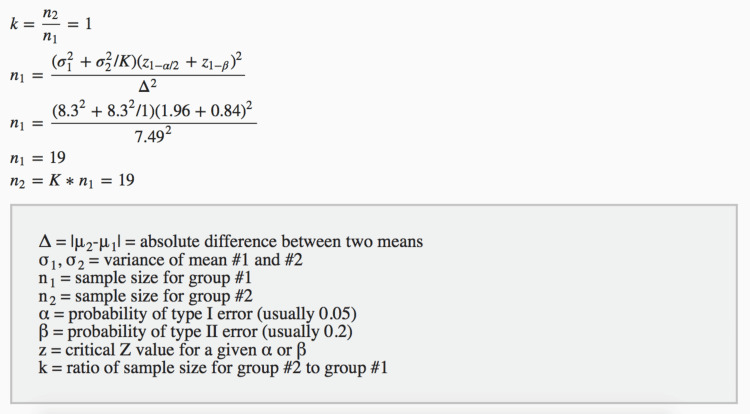
Sample size calculation. Based on salivary cortisol level values given in Table number 4 of the reference study by Goiato et. al., 2019 [15]; the minimum sample size for each group is 19, which is rounded to 20 in each group.

The research focused on 40 individuals diagnosed with type 2 diabetes mellitus who required complete dentures. The selection criteria were based on glycemic hemoglobin levels, and the participants were chosen from the Outpatient Department (OPD) of the Department of Prosthodontics, Sharad Pawar Dental College and Hospital, Wardha, India. The study spanned two years from October 2020 to October 2022. The formula for calculating the sample size (N = 2 s² (Z ß + Z/2)²/D², where N is the sample size, s is the edentulous population, and z is the coefficient of variance) ensured a robust and representative cohort for the investigation. Sample size calculation is explained in detail in Figure 1.

Prior to their involvement in the study, all participants provided informed written consent, underscoring the ethical imperative of voluntary participation. The study received approval from the Institutional Ethics Committee, with the reference number 2022-21/9390. A detailed and accessible presentation of the research and its procedures was given to participants in language tailored to their comprehension, ensuring clarity and understanding before the study commenced.

To ascertain the diagnosis of diabetes and establish glycemic control, hemoglobin A1C (HbA1C) measurements were conducted for all participants. The inclusion criteria specifically targeted fully edentulous patients with a minimum three-year diagnosis of type 2 diabetes who expressed a willingness to participate. Exclusion criteria were applied to individuals with additional systemic illnesses, bone and metabolic disorders, and other endocrine diseases, ensuring the study's focus and relevance.

Custom-made complete denture prostheses were crafted for each participant, addressing the unique requirements of both the maxilla and mandible. The Depression, Anxiety, and Stress Scale was employed to assess psychosomatic conditions associated with stress, anxiety, and depression. Participants were instructed to rate each statement based on its relevance to their experiences in the preceding week, using a scale from 0 to 3.

Saliva collection and measurement of salivary cortisol levels were integral components of the study. Participants were instructed to refrain from eating, drinking, or performing oral hygiene before and during the procedure. Saliva was collected in an unstimulated manner, allowing it to accumulate in the mouth before being spat into a graded container. Collected saliva samples were stored at -20 degrees Celsius until processed using a commercially available salivary cortisol enzyme-linked immunoassay (ELISA) test kit.

All tests, including HbA1c analysis, determination of depression, anxiety, stress-21 scale, and cortisol levels in saliva, were conducted in a standardized sequence one month after the insertion of complete dentures. Subsequent statistical analysis involved assessing data normality using a parametric test. Descriptive statistical analysis was then performed, and mean differences were examined using the independent t-test, maintaining a significance level of p < 0.05.

The statistical software utilized for data analysis was IBM SPSS Statistics for Windows, Version 24, (Released 2016; IBM Corp., Armonk, New York, United States). This rigorous approach to study design and execution ensured the generation of robust and reliable findings, contributing valuable insights into the intricate relationships among glycemic control, complete denture prostheses, and psychosomatic well-being in individuals with type 2 diabetes mellitus.

## Results

Analytical and descriptive statistics were completed. The standard deviation, mean, and median were used to describe the data. We looked at the normality of continuous data using the Shapiro-Wilk test. Since the data's distribution did not match a normal distribution, non-parametric tests were run to analyze the information. Spearman's rho correlation test was used to check the relationship between variables. The significance threshold was held at 0.05. A total of 40 edentulous patients, 24 males, and 16 females, participated in the study as presented in Table 1.

**Table 1:** Gender distribution among the study population. A total of 24 (60.0%) males and 16 (40.0%) females participated in the study.

Gender	N	%
Female	16	40.0
Male	24	60.0
Total	40	100.0

It was noted that the mean HbA1c readings varied statistically significantly before and after complete denture rehabilitation. The HbA1c values before rehabilitation (7.13 ± 0.63) were reduced to 6.53 ± 0.50 after complete denture rehabilitation as seen in Table 2.

**Table 2:** Comparison of HbA1c values before and after complete denture rehabilitation #P-value obtained using the Wilcoxon Signed Ranks Test; †p <0.05 indicates significance. SD: standard deviation

HbA1c	N	Mean	SD	Median	Z-value	P-value^#^
Before	40	7.13	0.63	7.00	-5.246	<0.001^†^
After	40	6.53	0.50	6.60		

Before and after complete denture rehabilitation, there was a statistically significant difference in the mean depression, stress, and anxiety ratings as presented in Table 3.

**Table 3:** Comparison of DASS-21 score before and after complete denture rehabilitation. #P-value obtained using the Wilcoxon Signed Ranks Test; †p <0.05 indicates significance. DASS-21: Depression, Anxiety, and Stress Scale - 21 Items; SD: standard deviation

Characteristics	N	Mean	SD	Median	Z-value	P-value^#^
Stress						
Before	40	13.02	2.43	14.00	-5.465	<0.001^†^
After	40	6.02	1.47	6.00		
Anxiety						
Before	40	11.77	2.45	12.50	-5.527	<0.001^†^
After	40	5.80	1.52	6.00		
Depression						
Before	40	12.80	1.85	13.00	-5.522	<0.001^†^
After	40	5.75	1.59	5.50		
DASS-21						
Before	40	75.25	7.50	75.00	-5.517	<0.001^†^
After	40	35.55	4.37	36.00		

Before and after complete denture rehabilitation, a statistically significant difference in DASS-21 score was discovered. After complete denture recovery, the mean Depression, Anxiety, and Stress Scale - 21 Items (DASS-21) score, which was 75.25 ± 7.50 at baseline, was lowered to 35.55 ± 35.55. There was a statistically significant variance in mean salivary cortisol concentration values before and after complete denture rehabilitation. The mean salivary cortisol concentration values at baseline (66.35 ± 30.54) were reduced to 31.22 ± 19.74 after complete denture rehabilitation as presented in Table 4.

**Table 4:** Comparison of salivary cortisol concentration values before and after complete denture rehabilitation. #P-value obtained using the Wilcoxon Signed Ranks Test; †p <0.05 indicates significance. SD: standard deviation

Salivary cortisol concentration	N	Mean	SD	Median	Z-value	P-value^#^
Before	40	66.35	30.54	13.00	-5.377	<0.001^†^
After	40	31.22	19.74	5.50		

**Figure 2:**
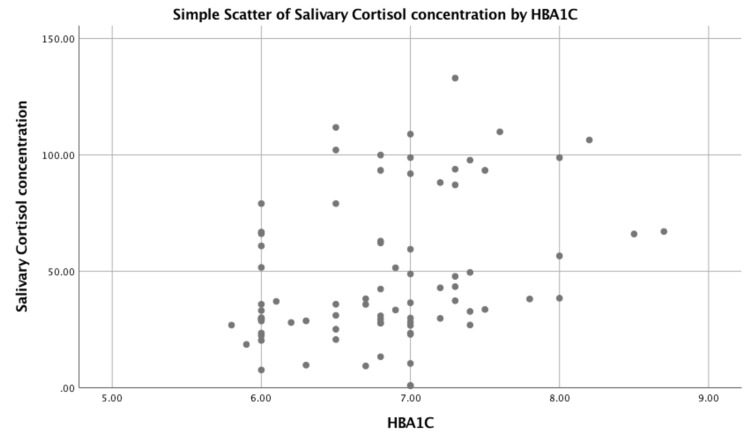
Graphical correlation of salivary cortisol concentration with HbA1c. HbA1c: hemoglobin A1c

**Figure 3:**
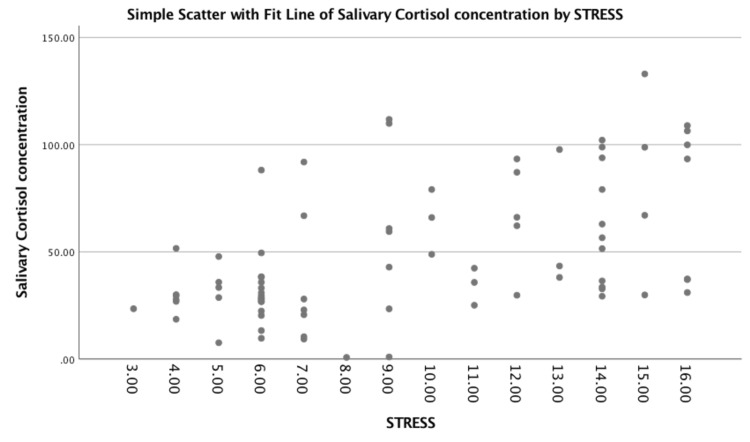
Graphical correlation of salivary cortisol concentration with stress.

The relationship between diabetes and salivary cortisol involves the intricate interplay of the endocrine system, stress response, and metabolic regulation. Cortisol is a steroid hormone produced by the adrenal glands in response to stress, and it plays a crucial role in regulating metabolism, immune function, and inflammatory responses. Stress, whether acute or chronic, triggers the release of cortisol. Individuals with diabetes may experience both physical and psychological stress related to managing their condition, which can contribute to elevated cortisol levels. Cortisol increases blood glucose levels by promoting gluconeogenesis (the production of glucose from non-carbohydrate sources) and reducing glucose utilization. In individuals with diabetes, this can exacerbate hyperglycemia. The HPA axis regulates cortisol production. In diabetes, especially in conditions like type 2 diabetes, there may be disruptions in this axis, leading to altered cortisol secretion patterns. Salivary cortisol measurement provides a non-invasive way to assess cortisol levels. Figure 2 shows the correlation of salivary cortisol concentration with HbA1c. Figure 3 shows the correlation of salivary cortisol concentration with stress.

Salivary cortisol is often considered a reliable marker of free, biologically active cortisol in the bloodstream. Some studies have explored the correlation between salivary cortisol levels and diabetes. Elevated cortisol levels, particularly in response to chronic stress, have been associated with insulin resistance and impaired glucose metabolism. Individuals with diabetes may exhibit variable responses to stress, impacting cortisol secretion. This variability can contribute to differences in glycemic control among individuals with diabetes. A statistically significant weak positive correlation (R = 0.365) was found between salivary cortisol concentration and HbA1c scores. A moderate positive correlation (R = 0.565) was found between salivary cortisol concentration and DASS-21 scores as seen in Table 5.

**Table 5:** Correlation of salivary cortisol concentration with other parameters. #P-value derived from Spearman's rho correlation test; †significant at p < 0.05

Salivary cortisol concentration	N	R-value	P-value^#^
HbA1c	40	0.365	0.001^†^
Stress	40	0.545	<0.001^†^
Anxiety	40	0.535	<0.001^†^
Depression	40	0.556	<0.001^†^
DASS-21 score	40	0.565	<0.001^†^

## Discussion

The study aimed to investigate the relationship between salivary cortisol levels, a stress biomarker, and the anxiety and tension experienced by type 2 diabetic patients undergoing rehabilitation with complete dentures. Both subjective and objective measures can be used to describe the accomplishment of conventional treatment of a complete denture. The quality of the denture, the state of the oral tissues, the patient's attitude and personality, the relationship between the clinician and patient, and socio-economic variables are all potential predictors of denture satisfaction. Furthermore, several studies conducted by various authors have indicated that those who are entirely edentulous may be more susceptible to co-morbidities, which include dementia, diabetes, asthma, cancer, and cardiovascular diseases. However, it is unclear whether these conditions are causal as a result of edentulism casual, in that the disease occurs as a result of aging. Thus, it is essential to assess the relationship between the general health of edentulous patients and complete denture rehabilitation from both a functional and a cosmetic perspective.

**Figure 4:**
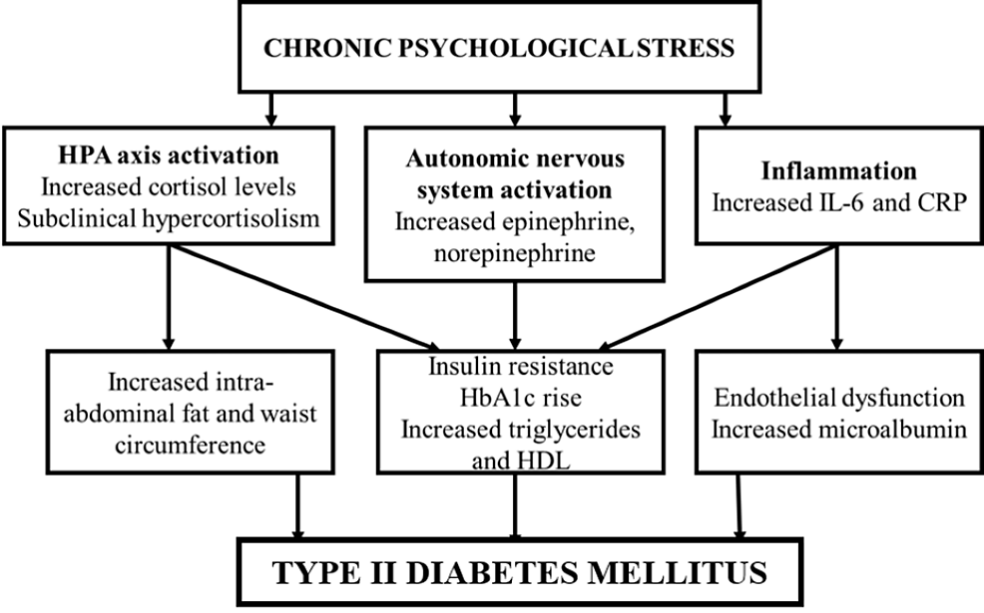
The bidirectional association between type-II diabetes, stress, depression, and anxiety.

This can be performed by assessing stress levels, utilizing salivary biomarkers, and examining their consequences on the overall health of the patient. Thanakun et al., 2014, studied 82 patients with metabolic syndrome who had a mean age of 48 years and a mean BMI of around 27.3 kg/m2 in comparison to 46 healthy individuals who had a mean age of 44.5 years and a mean BMI of 21.8 kg/m2 [16]. Harrison et al., 2014, conducted a study on twenty participants with diabetes mellitus and periodontitis; 21 systemically healthy participants suffering from periodontal disease and 21 healthy controls participated in the study [17]. HbA1c levels, tumor necrosis factor (TNF), and IL-6 levels in saliva and serum, as well as periodontal indices, were all reported. In the current study, salivary cortisol biomarkers were assessed, and HbA1c scores were evaluated for the severity of diabetes disease. The mean salivary cortisol concentration values before and after complete denture rehabilitation in controlled type 2 diabetes mellitus elderly patients were compared. From the results obtained from our study, a positive correlation (R = 0.365) was found between salivary cortisol concentration and HbA1c scores, suggesting salivary cortisol as a potential biomarker for the diagnosis of diabetes mellitus, as suggested in Figure 4.

Psychological stress has been linked to type 2 diabetes, according to Carroll et al., 2018; however, the exact mechanisms are still unclear [18]. In this study, subjects with and without diabetes participated in a controlled experimental comparison to determine if stress response is related to glucocorticoid and mineralocorticoid sensitivity. Psychophysiological stress testing was performed on 37 healthy controls and 37 diabetes patients. By inhibiting IL-6 levels caused by lipopolysaccharide (LPS) and prednisolone, respectively, glucocorticoid (GR) and mineralocorticoid (MR) sensitivity were determined. In terms of heart rate, blood pressure, MCP-1, IL-6, and stress recovery, people with diabetes displayed reduced stress responses.

The amount of cortisol excreted daily as well as baseline glucocorticoid sensitivity were both higher in people with diabetes. Before denture rehabilitation, the cortisol level in the saliva sample in the current investigation was statistically greater than the end level. Old, poorly adjusted, insufficient aesthetic prostheses, or those that hurt and impact the mucosa, change the patient's sense of self, raising stress and cortisol levels [19]. The findings revealed that comprehensive denture rehabilitation, after all necessary modifications and patient adaptation, had encouraged a decline in salivary cortisol levels.

All three components of DASS-21 scores, i.e., depression, anxiety, and stress, were positively correlated to the salivary cortisol concentrations, as found in the results of our study. This study is intended to compare and correlate anxiety with salivary cortisol levels in diabetes mellitus patients rehabilitated with complete denture prostheses. Although many similar studies have been done on dentate and partially dentate people, hospitalized geriatric patients with systemic and metabolic disorders, and people with symptoms of stress, anxiety, and depression [20], studies with salivary cortisol as a biomarker are rare in the edentulous diabetic population. We have also considered the DASS-21 scores in this study to assess anxiety, stress, and depression in our enrolled type 2 diabetic edentulous patients. Since our study has considered the salivary cortisol levels and DASS-21 scoring criteria in diabetic patients as biomarkers of stress and anxiety, it will add a good amount of literature to the existing research knowledge on cortisol as a diagnostic biomarker.

Also, our study depicts a lucid and appreciable decrease in the three parameters (i.e., salivary cortisol, HbA1c, and DASS-21 scores) after the insertion of a complete denture prosthesis in edentulate patients suffering from type 2 diabetes mellitus. Accordingly, there is a significant positive correlation present among DASS-21 scores and salivary cortisol range in diabetic patients following complete denture rehabilitation, which is further supported by our findings of a sharp decline in the amounts of the three study parameters in the diabetic edentulate population. 

## Conclusions

Salivary cortisol is a very sensitive biomarker because of its diurnal variation, which leads to dysregulation of the HPA axis. Reducing stress and anxiety results in lower salivary cortisol levels, thereby improving the quality of life of complete denture patients. Diabetes can also be best measured by monitoring HbA1c periodically. The study can be conducted with a larger sample size by combining other screening tools. Also, the study participants with different types of denture rehabilitation therapy can be applied and correlated for the same.
